# Plant economic strategies in two contrasting forests

**DOI:** 10.1186/s12870-023-04375-9

**Published:** 2023-07-22

**Authors:** Kuo Sun, Ruojun Sun, Yibo Li, Hongchao Ji, Bingrui Jia, Zhenzhu Xu

**Affiliations:** 1 State Key Laboratory of Vegetation and Environmental Change, Institute of Botany, Chinese Academy of Sciences, Beijing, 100093 China; 2 University of Chinese Academy of Sciences, Beijing, 100049 China; 3 Key Laboratory of Land Surface Pattern and Simulation, Institute of Geographic Sciences and Natural Resources Research, Chinese Academy of Sciences, Beijing, 100101 China

**Keywords:** Plant functional type, Leaf functional traits, Leaf economics spectrum, Leaf density, Alpine fir forest, Boreal coniferous forest

## Abstract

**Background:**

Predicting relationships between plant functional traits and environmental effects in their habitats is a central issue in terms of classic ecological theories. Yet, only weak correlation with functional trait composition of local plant communities may occur, implying that some essential information might be ignored. In this study, to address this uncertainty, the objective of the study is to test whether and how the consistency of trait relationships occurs by analyzing broad variation in eight traits related to leaf morphological structure, nutrition status and physiological activity, within a large number of plant species in two distinctive but comparable harsh habitats (high-cold alpine fir forest vs. north-cold boreal coniferous forest).

**Results:**

The contrasting and/or consistent relationships between leaf functional traits in the two distinctive climate regions were observed. Higher specific leaf area, photosynthetic rate, and photosynthetic nitrogen use efficiency (PNUE) with lower N concentration occurred in north-cold boreal forest rather than in high-cold alpine forest, indicating the acquisitive vs. conservative resource utilizing strategies in both habitats. The principal component analysis illuminated the divergent distributions of herb and xylophyta groups at both sites. Herbs tend to have a resource acquisition strategy, particularly in boreal forest. The structural equation modeling revealed that leaf density had an indirect effect on PNUE, primarily mediated by leaf structure and photosynthesis. Most of the traits were strongly correlated with each other, highlighting the coordination and/or trade-offs.

**Conclusions:**

We can conclude that the variations in leaf functional traits in north-cold boreal forest were largely distributed in the resource-acquisitive strategy spectrum, a quick investment-return behavior; while those in the high-cold alpine forest tended to be mainly placed at the resource-conservative strategy end. The habitat specificity for the relationships between key functional traits could be a critical determinant of local plant communities. Therefore, elucidating plant economic spectrum derived from variation in major functional traits can provide a fundamental insight into how plants cope with ecological adaptation and evolutionary strategies under environmental changes, particularly in these specific habitats.

**Supplementary Information:**

The online version contains supplementary material available at 10.1186/s12870-023-04375-9.

## Background

Understanding the relationships between plants and changing environment is essential in exploring ecosystem functioning responses to climate change [1–4]. Plants have evolved various intrinsic and extrinsic traits over an extended period of evolution and development. These traits are influenced by a combination of environmental and genetic factors, and they play a crucial role in shaping the behavior and function of plants [5, 6]. Actually, these functional traits explain the acquisition of resources in plants [7, 8] and reflect the trade-off between plant growth rate, leaf photosynthesis and the resource acquisition for long-term adaptations to environmental changes [9–11]. Therefore, analyzing leaf functional traits in different plants can help arrival at definitive mechanism responding to climate change and human activity. Previous studies using functional traits have broadly focused on testing plant defense synergy and antagonism [12, 13], predictions of climate change [14], and succession and composition of plant community [15], demonstrating the breadth of applications of functional traits. Although these studies include a number of plant species with multiple observations in many regions even globally [16, 17], the number of observations available for any given habitats is scant, especially with respect to the coverage in the two distinct plant communities. Thus, what and how leaf functional traits coordinate within a plant community, especially for the comparison between high-cold alpine vs. north-cold boreal forests, remains understudied.

Generally, the integrated whole-plant functional traits are divided into leaf functional traits, stem functional traits and root functional traits according to different organs of plants [18, 19]. Leaves are the primary organs for energy and material exchange in plants, and as a central aspect of plant behavior and function, are highly sensitive to environmental variations [20]. Therefore, leaf functional traits play a particularly important role in the relationships between plants and the environment, mainly including three components: leaf structural property, leaf nutrition status and leaf physiological activity [16]. Moreover, the leaf traits are closely related to each other and the relationship of traits can be expressed by leaf economics spectrum (LES) [3, 16, 17], which provides a general framework for carbon economics and nutrient use in leaves among all plant groups [16, 21]. However, there is still debate about whether LES can occur within a plant community in these distinctive but comparable harsh habitats [11, 22, 23].

Amongst leaf structural aspects, specific leaf area (SLA), an above-ground trait that is indicative of plant life history strategy along the fast-slow economics spectrum [19], represents the ability to utilize resources from environment and preserve the obtained resources, closely linking to plant survival strategies [24]. Leaf thickness (LT) and leaf tissue density (LD) have been considered vital functional traits due to their close linkage to SLA [25], reflecting the ability to acquire required resources and defense mechanism [26]. LT, as the important leaf shape component, can respond to the changes in light for photosynthesis and affect the energy and matter transformation in photosynthesis and water storage and utilization in leaves [27]. Study by Ryser [28] emphasizes that LD, as a pivotal trait for the ecological behavior of a species, was associated with plant growth rate, leaf life span and nutritional utilization patterns [28, 29].

Amongst leaf nutritious aspects, nitrogen, as the main components of enzymes in photosynthesis, is tightly related to the maximum photosynthetic efficiency [30, 31]. It also has a certain relationship with nutrient storage, which can reflect plant growth and physiological mechanism [31]. Amongst leaf physiological aspects, photosynthetic capacity, as the key plant traits determined by light harvesting and carboxylation reactions [32], was influenced by environmental factors mainly through the photosynthetic nitrogen use efficiency (PNUE), rather than through leaf nitrogen content [32]. The significant correlation between PNUE and SLA was also obtained; thus, PNUE has been regarded as an important leaf trait for determining N use efficiency, affecting the growth potential [33]. In addition, using LD, LT and PNUE should enrich our understanding the relationship between leaf structure and resource use [25]. However, how to use the three key traits in terms of LES remains under-studied, particularly under these extremely harsh environmental conditions in situ.

These leaf/plant functional traits closely linking to ecophysiological processes and production could be driven by biotic factors such as genetic variation (e.g., [10] ) and metabolite biosynthesis [34, 35], and abiotic factors such as soil nutrition [36–40] and soil pollution [41–43]. Leaf functional traits are largely reshaped by the local climate over millennia [4]. Therefore, quantifying the relationship between leaf functional traits and climate is the key to explaining what and how traits confer plants suitable for the specific climatic region. Westoby and Wright [44], based on global data, proved the urgent need for the studies in different geographical groups to obtain the precise patterns of leaf functional traits. However, the comparisons between specific climatic regions remains essentially unexamined. At present, climate change has seriously affected natural ecosystems, in particular in the high latitude and altitude of the northern hemisphere [45, 46]. It is worth mentioning that the two sites have the typical coniferous forest areas in China (i.e., the northern coniferous forest and the alpine coniferous forest, respectively), and have the cold temperature, but the causes of low temperature are totally different.

The Tibetan Plateau, is a unique geographical position with the highest plateau on Earth, belonging to the important ecological barrier of the Yellow River and Yangtze River basins. It has an important role in regulating the regional climate [47], and in maintaining the stability of ecological environment in China and even worldwide [47, 48]. Over recent decades, the Tibetan Plateau has undergone climate change, aggravated by human activities. For instance, the mean annual temperature (MAT) has increased by about 0.4 °C per decade since the 1970s which is twice that of the global average [49]. These climate fluctuations will strongly affect the Tibetan plateau ecosystem, changing the pattern, process and function of the local ecosystems [47, 48]. Due to its uniqueness and vulnerability of its geographical environment and ecosystems, the Tibet is an ideal place for the studies of global climate change [50], but it is the most underrepresented region in plant trait databases so far. Meanwhile, the Greater Khingan Mountains (GKM) is an important climate demarcation range in China, locating in the high latitude area of China. GKM has typical cold temperate vegetations, particularly the China’s only primeval cold-temperature coniferous forest with the largest area and best preservation. With the transitional nature of the geographical conditions as well as the effects of continental and marine monsoon, this region also become one of the most sensitive areas to climatic change [51]. MAT in this region will increase by 2.3-5.0 °C and the mean annual precipitation (MAP) will increase by 72–164 mm at the end of the 21st century.

Under global warming, in some specific regions, especially in high latitude [52] and high altitude [53] areas, rising temperatures were significantly higher than the global average [52, 53]. While vegetation, as the most active component of terrestrial ecosystems, responds rapidly to climate change, and the pattern of leaf functional traits in the particular environment largely reflects the focal properties of the ecosystem [3, 17]. Previous studies also showed that the relationships between leaf functional traits have different response patterns and trade-offs among climate types, suggesting that environmental factors and genetic traits affect leaf trait relationships in plants [54, 55]. It implies that LES may occur and differ in both contrasting ecosystems: high-cold alpine and north-cold boreal forests. Although the climate has some similar features due to the lower temperature, it is unknown whether the adaptive strategies differ in two contrasting forests, high-cold alpine forest vs. north-cold boreal forest. Plants may prosper under a moderate warming condition [14, 56, 57] suggesting plant species would place on the resource-acquisitive side in north-cold boreal forest due to the higher temperature during the peaks of the growing seasons (11.6℃ in alpine forest vs. 16.2℃ in boreal forest) (Table S1).

A body of research has comprehensively analyzed leaf functional traits on different vegetation types and plant functional types (PFTs) (e.g., [33, 58–60] ). It is indicated that evergreen xylophyta are often more slow-growing and resource-conservative, while herbs tend to be more fast-growing and resource-acquisitive [61–63]. Thus, leaf functional traits may markedly vary and the strategies of plants adapting to environmental factors would differ between PFTs (PFTs, e.g., [33, 62, 64] ). The relationships among leaf functional traits might be divergent between herb and xylophyta groups in both alpine vs. boreal forests.

This study was designed to explore patterns of relationships between leaf functional traits in the two geographical locations (both high-elevation and high-latitude) with uniqueness and sensitivity to environments. From a plant functional ecology perspective, these insights provide a better understanding of the existing relationship between plant functional traits in different climate types. Three hypotheses were suggested: (1) LES can be tested in both high-cold alpine and north-cold boreal forests, and the strong but contrasting relationships between leaf functional traits in the two distinctive climate regions could occur; (2) Based on LES and resource acquisition–conservation theories, plant species would cluster on the conservative side in high-cold alpine forest, whereas plant species may cluster on the acquisitive side in north-cold boreal forest; (3) Divergent relationships between leaf functional traits between herb and xylophyta groups may appear at each site. The major objects of the current results are also to test whether and how the divergent and/or convergence of leaf functional traits exists in the both forests. This can inform the relevant classic ecological theories in the two distinct plant communities—high-cold alpine vs. north-cold boreal forests under the both extremely harsh environmental conditions.

## Results

### Changes in leaf functional traits in two contrasting ecosystems

**Fig. 1:**
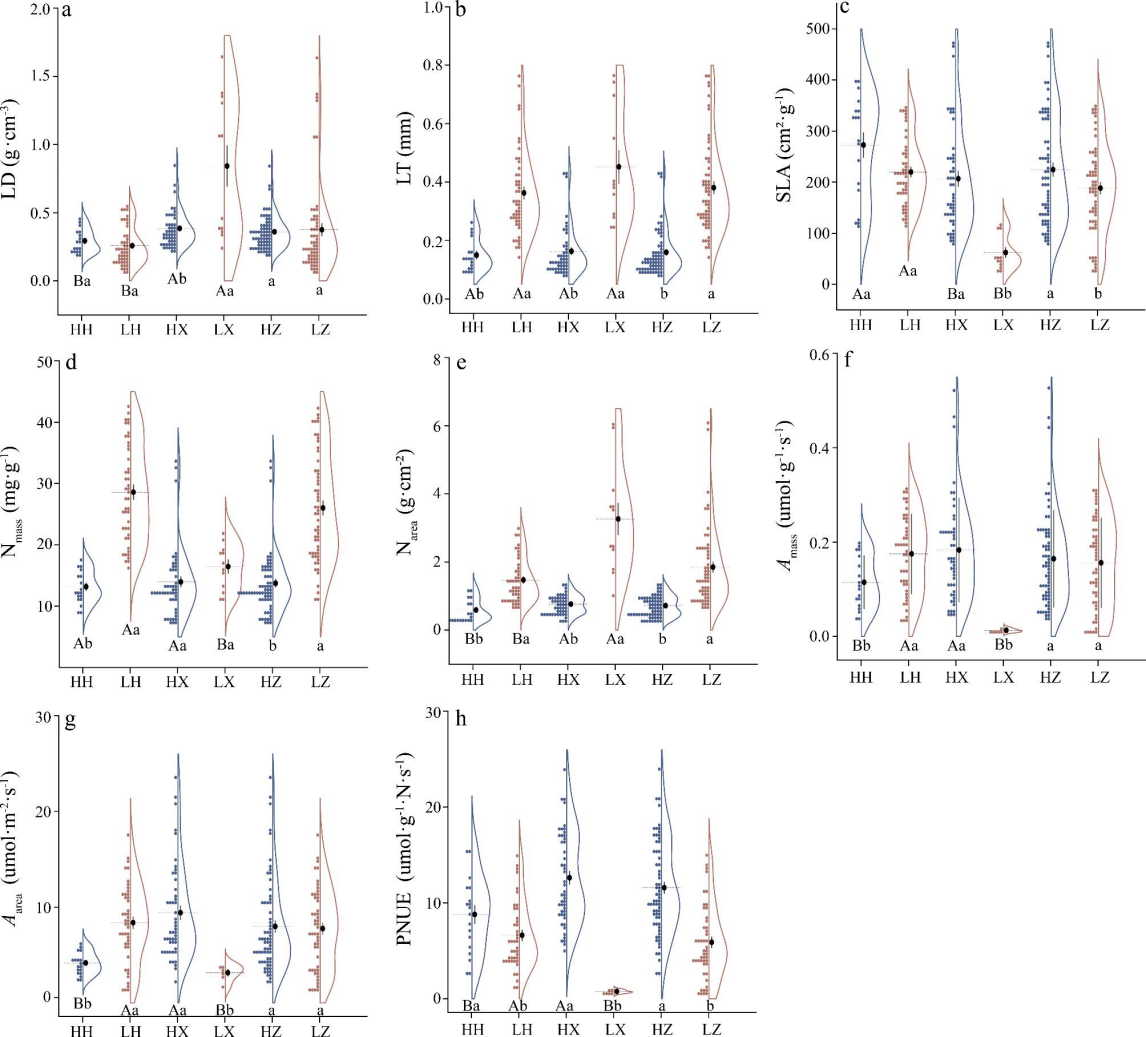
Changes in leaf functional traits at the two sites. Points and density curves represent data distribution of leaf functional traits in herbs at Huzhong (HH) and Linzhi (LH), in xylophyta at Huzhong (HX) and Linzhi (LX), and across the two plant functional types at both Huzhong (HZ) and Linzhi (LZ) sites. Leaf functional traits included leaf tissue density (LD, **a**), leaf thickness (LT, **b**), specific leaf area (SLA, **c**), leaf nitrogen concentration per unit mass (N_mass_, **d**), leaf nitrogen concentration per unit area (N_area_, **e**), light-saturated photosynthetic rate per unit mass (*A*_mass_, **f**), light-saturated photosynthetic rate per unit area (*A*_area_, **g**), photosynthetic nitrogen use efficiency (PNUE, **h**). Black points denote means with SD bars. Different capital and lowercase letters indicate significant differences among plant functional types and sites, respectively (*p* < 0.05)

The functional leaf traits at the two sites are shown in Table S1. Overall, a broad of variation in eight leaf economic traits was observed between plant functional types (PFTs, i.e., herb vs. xylophyta) at the two distinctive habitats (i.e., north-cold boreal forest vs. high-cold alpine forest). Across PFTs, the variations of all leaf traits between the two sites differed significantly, except LD, *A*_mass_ and *A*_area_ (Fig. 1, Table S2). For herbs, the significant differences in all traits between the two sites were obtained except LD and SLA. For xylophyta, no significant difference in N_mass_ between Huzhong and Linzhi was found. Higher values of SLA, *A*_mass_, *A*_area_ and PNUE were obtained in Huzhong than in Linzhi (Fig. 1, Table S2). When two PFTs were compared across the two sites, herbs had higher SLA, lower LD, LT and N_area_ than xylophyta. Herbs in Huzhong were found to have lower N_mass_, PNUE and photosynthetic rates (in terms of *A*_area_ and *A*_mass_) than those in xylophyta, whereas the opposite trend of these traits occurred in Linzhi—herbs had larger values of the traits than xylophyta (*p* < 0.05).

According to the analyses on coefficient of variation, generally, the traits in Linzhi were slightly more variable than those in Huzhong (Table S2). N_mass_ showed the least variation under the climate of Linzhi (CV = 33.68%). LD was the greatest variable (CV = 92.64%) in Linzhi, while it proved the least variation in Huzhong (CV = 34.83%). In summary, LD, N_area_ and PNUE were distinctly different traits between the two sites, while both *A*_area_ and *A*_mass_ differed slightly.

### Relationships between leaf functional traits

**Fig. 2:**
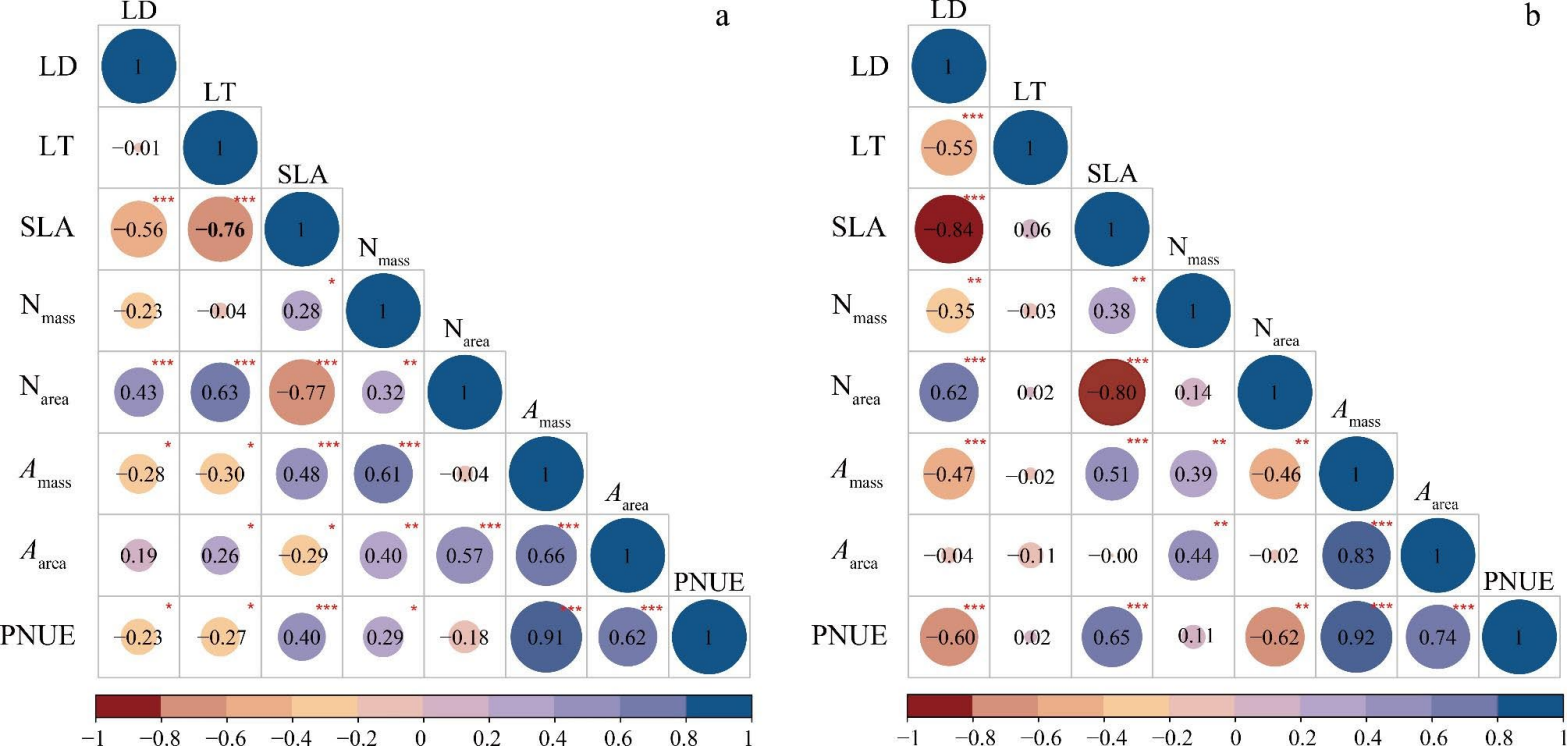
Correlations between leaf functional traits at Huzhong (**a**) and Linzhi (**b**) sites. *, *p* < 0.05; **, *p* < 0.01; ***, *p* < 0.001. For abbreviations, see Fig. 1

According to Spearman’s correlation coefficients, most of the functional traits at two sites were significantly correlated with each other (Fig. 2). In Huzhong, SLA, N_area_, *A*_mass_ and PNUE were significantly correlated with LD; meanwhile significant relationships of LT, SLA, N_mass_, N_area_, *A*_mass_ and PNUE with LD were observed in Linzhi (Fig. 2). N_area_ was positively correlated with *A*_area_ in Huzhong, but it negatively correlated with *A*_mass_ and PNUE in Linzhi. In addition, there were strong correlations between LD and SLA, *A*_mass_/*A*_area_ and N_mass_, SLA and PNUE in both climatic zones. LT has strong relationships with SLA, N_area_, *A*_mass_, *A*_area_ and PNUE in north-cold boreal forest; whereas it did not significantly relate to any traits except LD in high-cold alpine forest. The results displayed the contrasting and/or consistent relationships between leaf functional traits in the two distinctive climate regions (north-cold vs. high-cold habitats), highlighting that the coordination and/or trade-off may occur (Fig. 2, Table S2-S5).

**Fig. 3:**
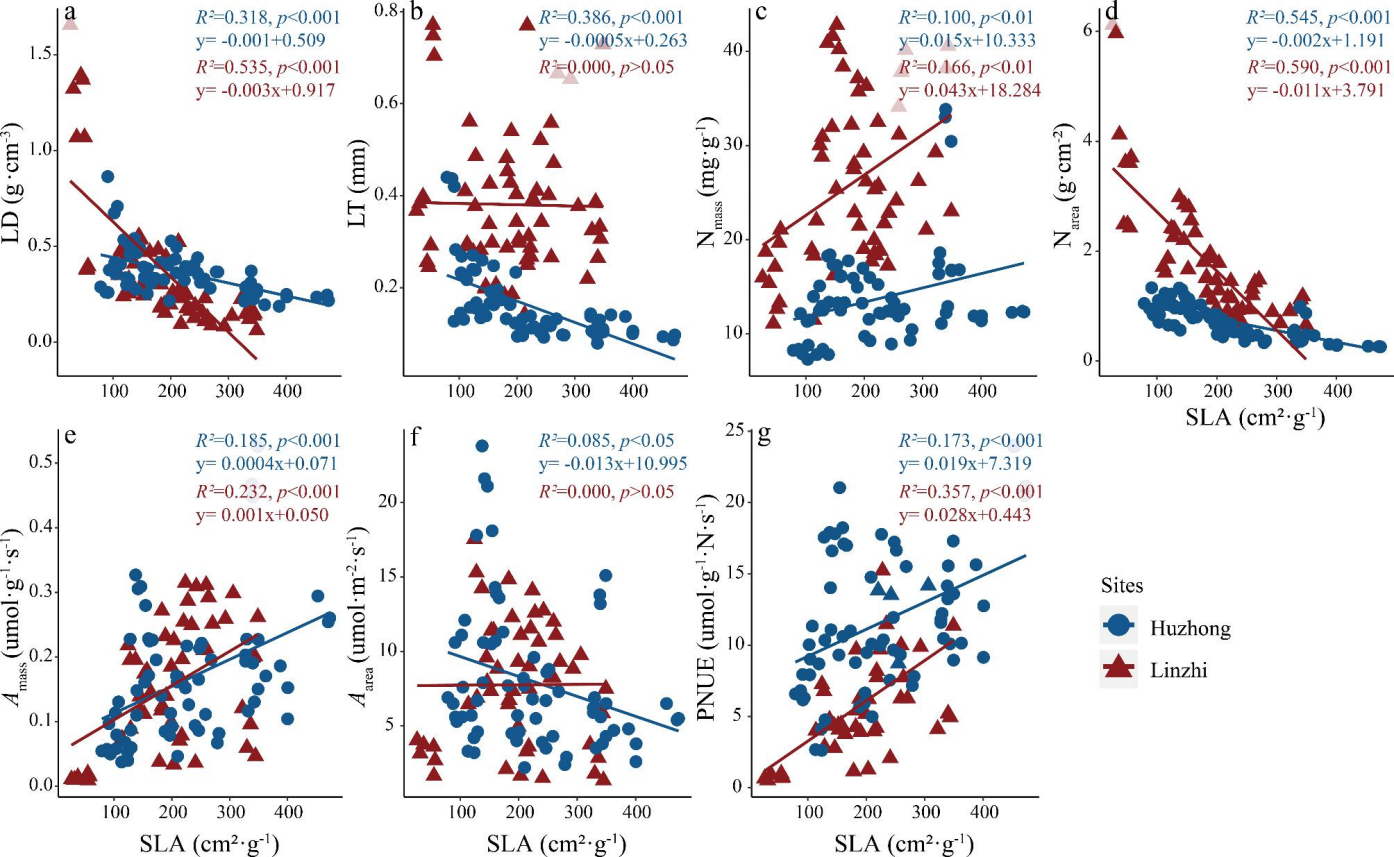
Regressive relationships of SLA with other leaf functional traits. For abbreviations, see Fig. 1

The associations of leaf functional traits with SLA are shown in Fig. 3. Linear negative relationships were observed between SLA and LD and N_area_, while SLA was significantly positively correlated with N_mass_, *A*_mass_ and PNUE. The slopes of the linear relationships of SLA with LD, N_mass_, N_area_, *A*_mass_ and PNUE in Linzhi were significantly steeper than those in Huzhong (Table S4), indicating that the LD, N_mass_, N_area_, *A*_mass_ and PNUE of alpine vegetation were more sensitive to SLA. By contrast, the slope between SLA and LT in the alpine climate was significantly negative more than the slope in the cold temperate climate.

**Fig. 4:**
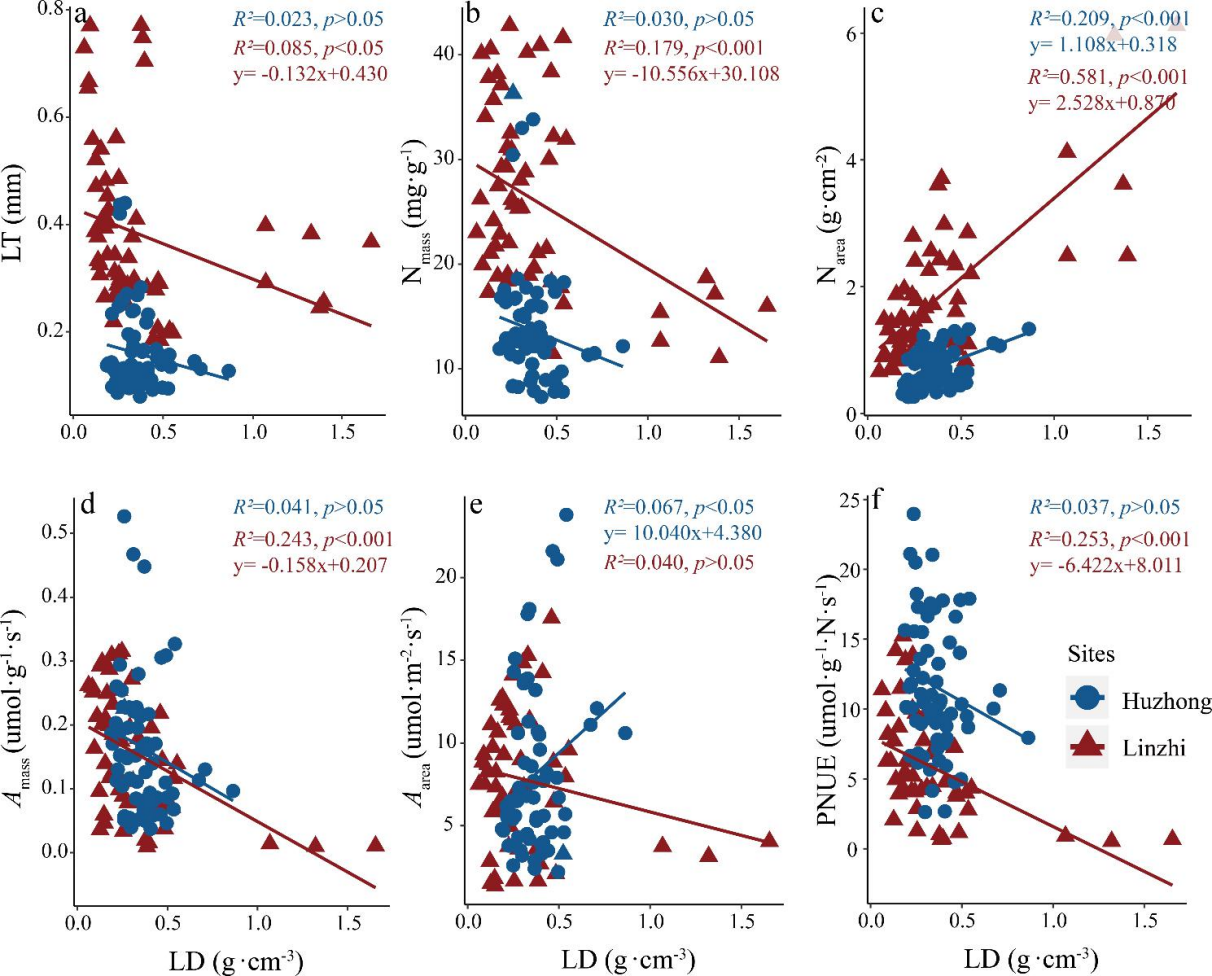
Regressive relationships of LD with other leaf functional traits. For abbreviations, see Fig. 1

When the regressions of LD against other traits at two sites were tested (Fig. 4), N_area_ significantly increased with LD with a higher slope in Linzhi than Huzhong (Fig. 4c). LD were significantly negative correlations with other leaf functional traits at Linzhi site (i.e., high-cold alpine area) except N_area_ and *A*_area_. However, positive significant relationship between *A*_area_ and LD occurred in the north-cold boreal forest (Fig. 4e).

**Fig. 5:**
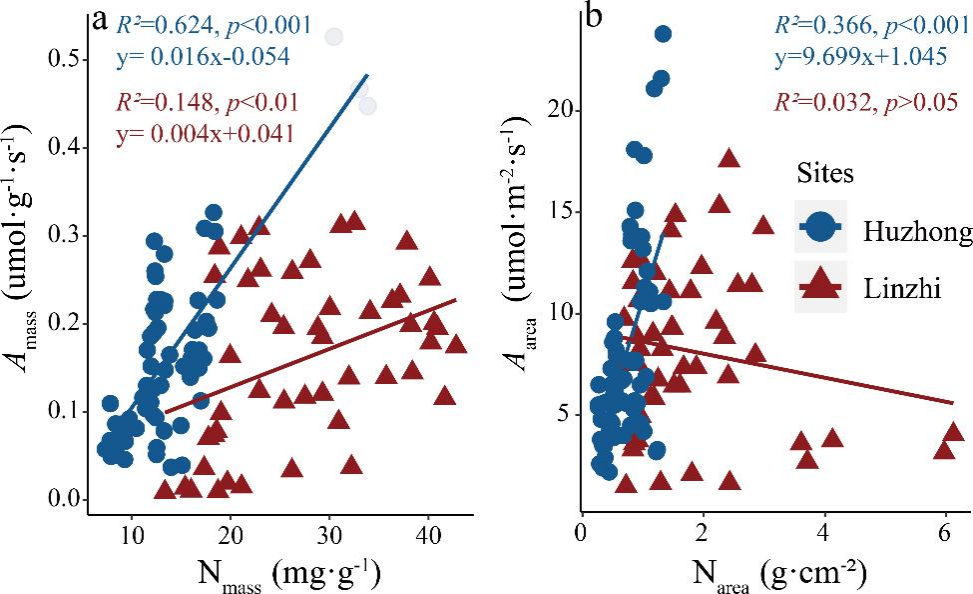
Regressive relationships between leaf N content (N_mass_ and N_area_) and light-saturated net photosynthetic rates (*A*_mass_ and *A*_area_)

As shown in Fig. 5, there were different relationships between leaf nitrogen content (both N_mass_ and N_area_) and photosynthetic-related traits (i.e., both *A*_mass_ and *A*_area_). With the regression analysis, N_mass_ showed a clear positive correlation with *A*_mass_, and the slope was significantly higher in Huzhong than in Linzhi. *A*_area_ significantly linearly increased with N_area_ in Huzhong but not in Linzhi. Significant differences in the values of both slopes and intercepts were found between high-cold alpine and north-cold temperate vegetation in two climatic zones (see Figs. 3, 4 and 5, Tables S4-S5). Furthermore, the intercept values of relationships between leaf structural traits (SLA, LD) and leaf nutrient traits (N concentrations on both mass and area basis) were greater in Linzhi than Huzhong; however, generally, there were greater intercepts of relationships of SLA and LD with photosynthetic rate (both *A*_area_ and *A*_mass_) and PNUE in Huzhong than in Linzhi (Table S4). It again highlighted the dependence on ecological habitats when characterizing the relationships between the functional traits.

### Principal component analysis on functional traits

**Fig. 6:**
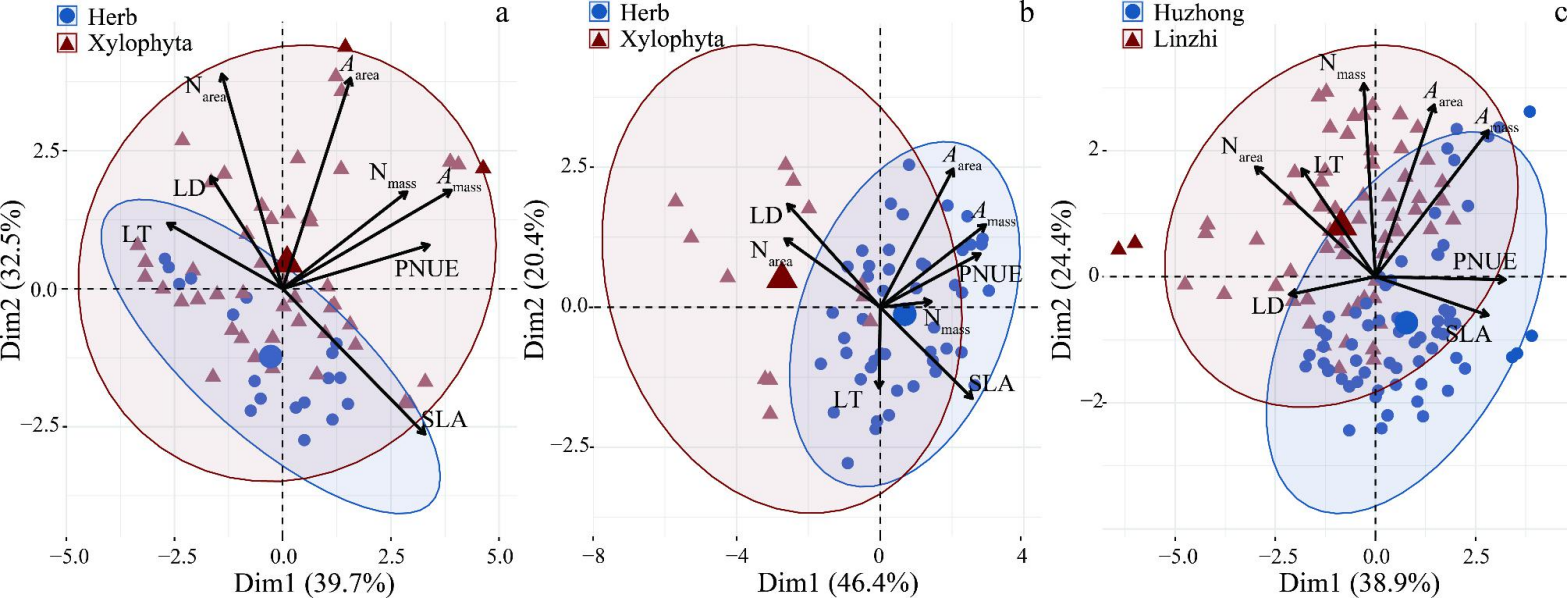
Principal component analysis (PCA) on plant functional traits for the two plant functional types (PFTs, i.e., herbs and xylophyta) at Huzhong (**a**) and Linzhi (**b**) sites, and across the two sits (**c**). Dim 1 and 2 represent PC factor 1 and PC factor 2, respectively. For abbreviations, see Fig. 1

The loading plot from principal component analysis (PCA) shows the pattern and relationships among leaf traits according to life forms (PFTs). In the Huzhong area, there was a distinct distribution of herb along PC1 (Fig. 6a), whereas xylophyte tended more towards the PC2. However, the opposite distribution pattern was observed in Linzhi (Fig. 6b). It again indicated the contrasting distribution difference between Huzhong and Linzhi sites. The loading variation of multiple traits along the PCA axes revealed the resource utilization strategies of vegetation at the two climate types (Fig. 6). At both Huzhong and Linzhi sites, the first two principal components accounted for 72.20% and 66.80% of total trait variation, respectively (Fig. 6a and b). The first component scores at two sites were significantly different (Table S6). PCA demonstrated divergent patterns in some traits at both Huzhong and Linzhi sites: *A*_mass_ was the main contributor to the first component, followed by PNUE and SLA in Huzhong (Fig. 6a). However, *A*_mass_ was the major contributor to PC1, followed by PNUE and N_area_ in Linzhi. At both Huzhong and Linzhi sites, PC1 and PC2 explained 38.90% and 24.40% of variation of total traits, respectively (Fig. 6c). The first component ran from conservative traits (low SLA and high LD) to acquisitive leaves (high SLA and low LD). This finding supported the hypothesis 1 and 2, that is, high-cold alpine and north-cold species could manifest divergent resource acquisition strategies.

### Structural equation modeling for the causal relationships

**Fig. 7:**
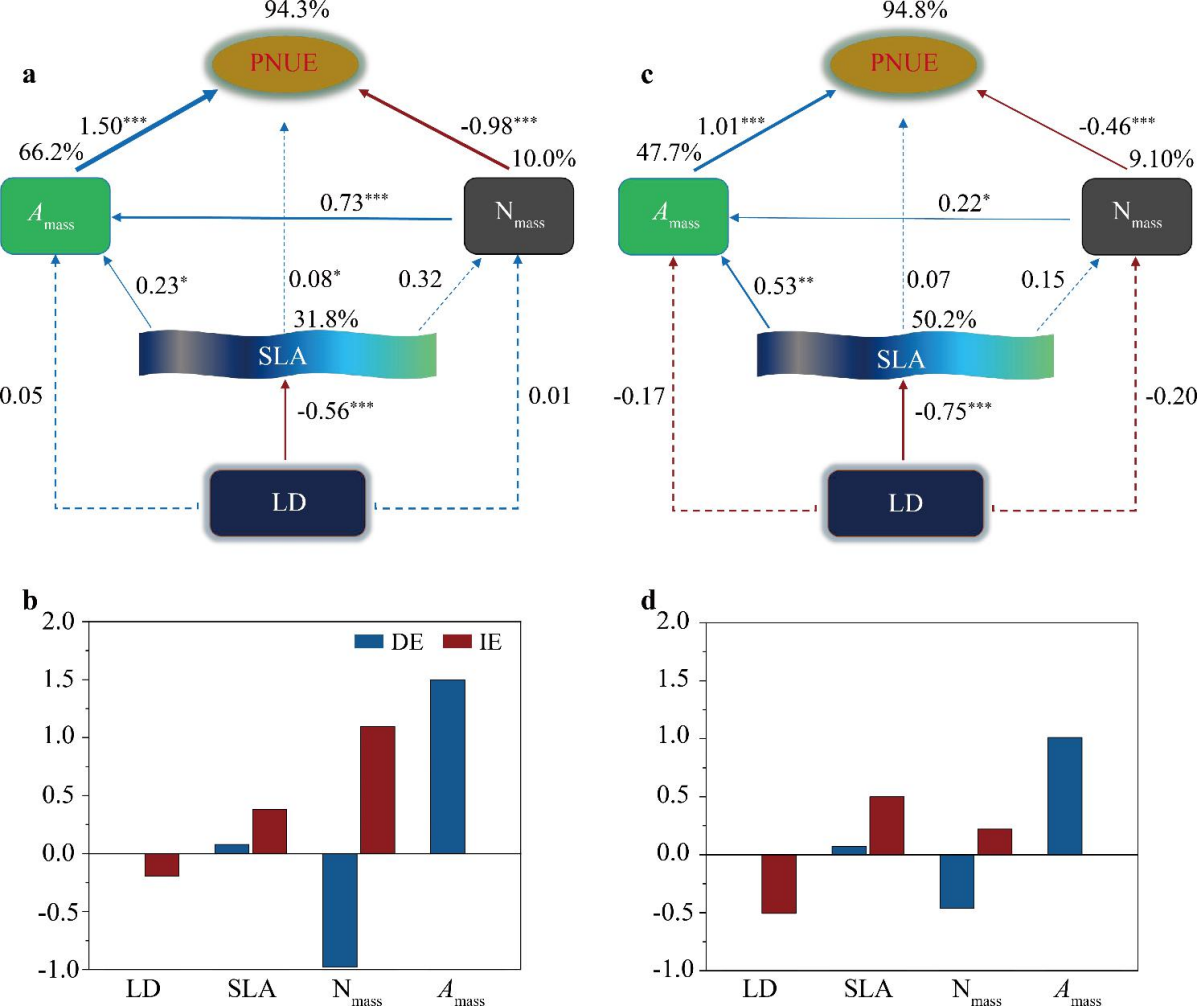
Structural equation modeling (SEM) on key leaf functional traits at Huzhong (**a, b**) and Linzhi (**c, d**) sites. Direct (DE) and indirect effects (IE) are given at both Huzhong (**b**) and Linzhi (**d**) sites, respectively. Solid blue and red arrows represent significant positive or negative relationships at *p* < 0.05 levels, whereas dashed blue and red arrows represent no significance (*p* > 0.05). Values above arrows indicate the standard path coefficients, and their significances at 0.05, 0.01, and 0.001 levels are marked by *, **, and ***, respectively. Percentages on rectangles indicate the variance explained by the models. For abbreviations, see Fig. 1; and for SEM statistical information, see Table S7

Based on the dominant functional traits, structural equation models (SEMs) were performed to further reveal the regulatory mechanisms of the combined factors affecting plant species in high-cold alpine forest (Linzhi) and north-cold boreal forest (Huzhong) (Fig. 7). In both Huzhong and Linzhi areas, LD and SLA were significantly negatively correlated with a path coefficient of -0.75 in Linzhi, and that of -0.56 in Huzhong, indicating a stronger response of SLA to LD in Linzhi. LD had weak direct relationships with *A*_mass_ and N_mass_ at the two sites. Significant relationships between SLA and *A*_mass_ occurred at both Huzhong and Linzhi sites (path coefficients of 0.23 and 0.53, respectively), with closer relationship in Linzhi region. At Huzhong site, N_mass_ and *A*_mass_ showed a strong positive correlation (Fig. 7a); similarly, significant correlation was observed in Linzhi (Fig. 7b). SLA directly but slightly affected PNUE (0.08) in Huzhong, but no link between them was found in Linzhi. For the relationship among the photosynthetic traits, these significant effects were also observed: PNUE was strongly positively affected by *A*_mass_ but significantly negatively affected by *N*_mass_. They were more closely related to PNUE in Huzhong than in Linzhi. LD indirectly affected PNUE via mediating SLA, *A*_mass_ at both sites with high explanation rates of 94.3% and 94.8% at Huzhong and Linzhi sites, respectively (Fig. 7).

## Discussion

### Patterns of leaf functional traits between PFTs

As introduced above, leaf functional traits and their relationships depend on vegetation types and functional types [58, 60], Evergreen xylophyta plants often have more slow-growing and resource-conservative strategies, meanwhile herb species might be more fast-growing and resource-acquisitive [61–63]. These results proved the marked differences in leaf functional traits and the strategies of plants that adapt to the environmental changes between plant functional types (PFTs) [33, 62, 64]. The current results underline the coordinated changes in key leaf functional traits in the north-cold boreal vs. high-hold alpine habitats under the two distinctive extreme harsh conditions.

Among the different PFTs presented in this study, we found that herbs had smaller LD and LT than xylophyta. Due to plant competition, the lower LD can maximize the photosynthetic rates and benefit plants in cool, wet or shaded environmental conditions [65]. The potential physiological mechanisms involved may be: the leaves with smaller LD and LT often have less numbers of mesophyll cell layers and lower cell mass densities [66]. They could favor the spread of CO_2_ in mesophyll cells, weakening the resistance to gas exchanges and subsequently increasing photosynthetic rates [29, 67]. Thus, the short herbaceous plants can increase carbon investment by reducing LT to obtain more light, water and nutrients to improve photosynthetic capacity, finally promoting plant growth [65]. Conversely, the xylophyta species with larger LD may hinder the spread of CO_2_ in mesophyll cells, consequently increasing the resistance to gas exchanges [29].

SLA can be used as a reliable indicator of carbon acquisition ecological strategies [3, 68]. In our study, SLA tended to be larger in herbs than in xylophyta, consistent with those previous reports [69, 70]. The marked differences of light conditions between the two plant groups might explain this phenomenon [69, 71, 72]. Herbs are often in lower layers in the whole community under less light condition owing to the shading of upper leaves [71]. In order to adapt to this low light, plants increase SLA to promote the absorption of light and ensure quickly acquire resources and high growth rate [72]. Thus, the ecological strategies employed by herb groups tend to be resource-acquisitive (e.g., [68, 73] ). While in the upper layers in the whole community, xylophyta is irradiated by strong light, leading to a rapid water transpiration. Xylophyta could tend to reduce SLA to minimize the water loss and enhance the preservation of resources to reduce the photosynthetic damage [74]. This makes more nutrients to construct the leaf cytoderm and vascular tissue, which help xylophyta adapt to the environmental changes [75, 76].

Previous results have indicated that high nutrient and photosynthetic capacity as well as resource use efficient (e.g., PNUE) often occur in herbaceous plants rather than xylophyta group [16, 61]. The fast growth needs more nutrients including N_mass_, resulting in the higher N in herbs than slow-growing and resource-conservative woody trees [61, 63]. Enhanced pant growth clearly requires maximizing photosynthetic capacity at a given leaf nitrogen content level [77]. Species such as herbs in the shade partition could allocate more nitrogen into photosynthetic apparatus such as the thylakoids [77, 78], leading to a higher PNUE. This process may relate to plant species evolution or domestication [79]. On the contrary, the xylophyta is especially required to be more tolerant to adverse effects of high light than herbaceous plants, thus the leaf N is mainly used to construct mesophyll cells, resulting in less nitrogen to photosynthetic organs and weakening photosynthetic capacity (*A*_mass_, *A*_area_ and PNUE) [80]. Moreover, in the current study, N_area_ was higher in xylophyta than in herbs, this result also is in line with a conventional report by Wright et al. [16]. Xylophyta might reduce their SLA by increasing dry mass of leaves per unit area, leading to larger N_area_ to resist to high light. However, these could be confirmed only at Linzhi rather than Huzhong by our results (Fig. 1d-h). In the north-cold boreal forest, leaf N concentration, photosynthetic capacity and PNUE were significantly higher in xylophyta than herb plants, which may be due to the different solar radiation intensities in both upper and bottom leaves in the both two distinctive regions. All of these results indicated that the responses of leaf functional traits to environmental conditions in these extreme harsh regions, including extremely high latitude and extremely high altitude areas, might be closely associated with PFTs [64, 81]. This largely confirmed our third hypothesis. However, these physiological processes and the underlying mechanisms on the relationships between photosynthesis and resource use for the different PFTs such as xylophyta and herb groups need further to be explored (e.g., [78, 79, 82] ).

### Correlations between key leaf function traits

Reich et al. [83] suggested that the similarity of coordinated relationships in disparate habitats can reflect convergent evolution. Our study indicated that most of the leaf functional traits were closely related to each other (Figs. 2, 3, 4 and 5). There were strong correlations between leaf N (N_mass_) and photosynthetic capacity (*A*_mass_) at both sites, but not between N_area_ and *A*_area_ at Linzhi site (Fig. 5). It is possible that N distribution in a whole leaf and other limiting factors such as stomatal features on photosynthesis affect their relationships [77, 78, 84]. In addition, the *A*_mass_-N_mass_ relationships seemed to show a climate-related tend, with a higher slope in the boreal climate than in the alpine climate, indicating the high sensibility of photosynthetic activity to leaf N in Huzhong relative to Linzhi (Fig. 5). In addition, this variation could also be distinguished by PNUE between both the areas. This implies that the allocation of leaf nitrogen into photosynthetic apparatus may strongly depend on climate zones [77, 78, 85].

Similarly, along environmental gradients, leaf functional trait relationships in different plant functional groups can show a broad range of the variations in their slope/intercept values, possibly reflecting different resource allocation [86, 87]. In the current study, these slope differences could show the diversity in survival strategies between the two regions. For the relationships of SLA against other traits, most of the slopes in alpine regions were greater than those in boreal regions (Fig. 3), indicating that other functional traits is more sensitive to SLA in the alpine ecosystem. The intercepts were larger in the relationships between SLA and leaf structure and nitrogen concentration, but smaller in the relationships with photosynthetic rates. It suggested that the association of leaf structure with N nutrient may be strongly constrained by intrinsic factors such as the genetic background and elemental metabolism in plants to exhibit functional convergence.

Based on PCA analysis, we found that these photosynthetic traits were convergent more in north-cold boreal forest than in high-cold alpine forest. It implies that the former would exert a joint response to local environmental change [88]. Additionally, the results from SEM were partially inconsistent with those using linear regressions in our study. It may be that the combined/interactive effects of/between the multiple traits with SEM are not same patterns from the effects of single factors each other. Nevertheless, plant growth is jointly controlled by physiological processes and environmental factors, which closely related to their relationships among traits and environmental variables. By natural selection, over a long-term span, an optimal combination of plant functional traits would be selected to adapt to external environment, which also reflects the ecological strategies adopted by plants in a distinctly given habitat [89, 90].

### Resource acquisition strategies in high-cold alpine vs. north-cold boreal forests

LES could link to the classical trade-off theory based on relationships among leaf functional traits, allowing us to understand the adaptive strategies of plants between resource acquisition and conservation [16]. Our results indicated that the leaf functional traits in north-cold boreal forest were largely distributed in the resource-acquisitive strategy spectrum, a quick investment-return behavior; while those in the high-cold alpine forest tended to be mainly placed at the resource-conservative strategy end, a slow investment-return phenomenon. The largely confirmed our first and second hypotheses. We found that the coordinated relationships among leaf functional traits measured in two forest were ranged within global LES patterns [16], also supporting the convergent evolution hypothesis in terms of co-variation in plant functional traits [19, 87, 91].

It is found that the most significant difference between the two climate types was that LD, LT, N_mass_ and N_area_ were lower in boreal forest than in alpine forest (Fig. 1). In addition, SLA and PNUE were higher in the north-cold boreal forest than in high-cold alpine vegetation. The low tissue density could be responsible for the quick acquisition of resource in north-cold boreal forest [25, 28]. While in the alpine ecosystem, leaves may tend to be higher LD [25], suggesting the low *A*_mass_ may be a result of constraints made by tissue structure [92] (Fig. 1f). Furthermore, increasing secondary metabolism activity caused by higher light density may explain this phenomenon [93]. The reduced LT in boreal ecosystem confirmed here was probably due to decreased palisade development [94]. Conversely, an increase in LT is often used to improve the anti-interference ability and energy reservation to cope with extremely high light irradiance [25]. The present results showed that leaf N concentrations were higher in alpine vegetation than in boreal vegetation. This may be due to the selection of the measured species. Previous studies have shown that evergreen plants usually have lower leaf N [30, 95]. Most species measured in the boreal forest are evergreen species. In addition, studies on alpine plants have revealed that leaf N concentration usually increases with increasing elevation [64, 96, 97]. Maire et al. [98] found that leaf N was more affected by the joint effects of soil and climate, and the soil N content was higher in alpine forest than in boreal forests [99].

At global scale, SLA varies significantly in different regions [2]—it increases with latitude [100], but decreases with elevation [101]. These results are consistent with our findings in both boreal vs. alpine plant species. The plants in north-cold boreal forest may enhance photosynthetic capacity to adapt the relatively less light by increasing SLA; meanwhile plants in high-cold alpine forest might regulate stomata to constrain excessive light energy by decreasing SLA [102]. All of these can improve the tolerance to adverse habitats such as low temperature and high radiation [103]. Moreover, species with high SLA almost invariably have a high PNUE. It may be explained mainly by the fact that high-SLA species allocated more N to Rubisco [33]. Therefore, higher PNUE was observed in north-cold boreal forest than in high-cold alpine forest. While the lower PNUE in alpine forest is because its unique geographical conditions that is characterized by high altitude, low temperature and high UV-B radiation [97, 104]. This also lead to lower photosynthetic rates [81](Fig. 1f-h). A report by Ali et al. [32] also showed that species from higher latitude zones tend to have high photosynthetic capacity. These again reflects a resource acquisition–conservation economics spectrum [19] in the alpine plant communities. These all support the results presented in our study (Fig. 1).

**Fig. 8:**
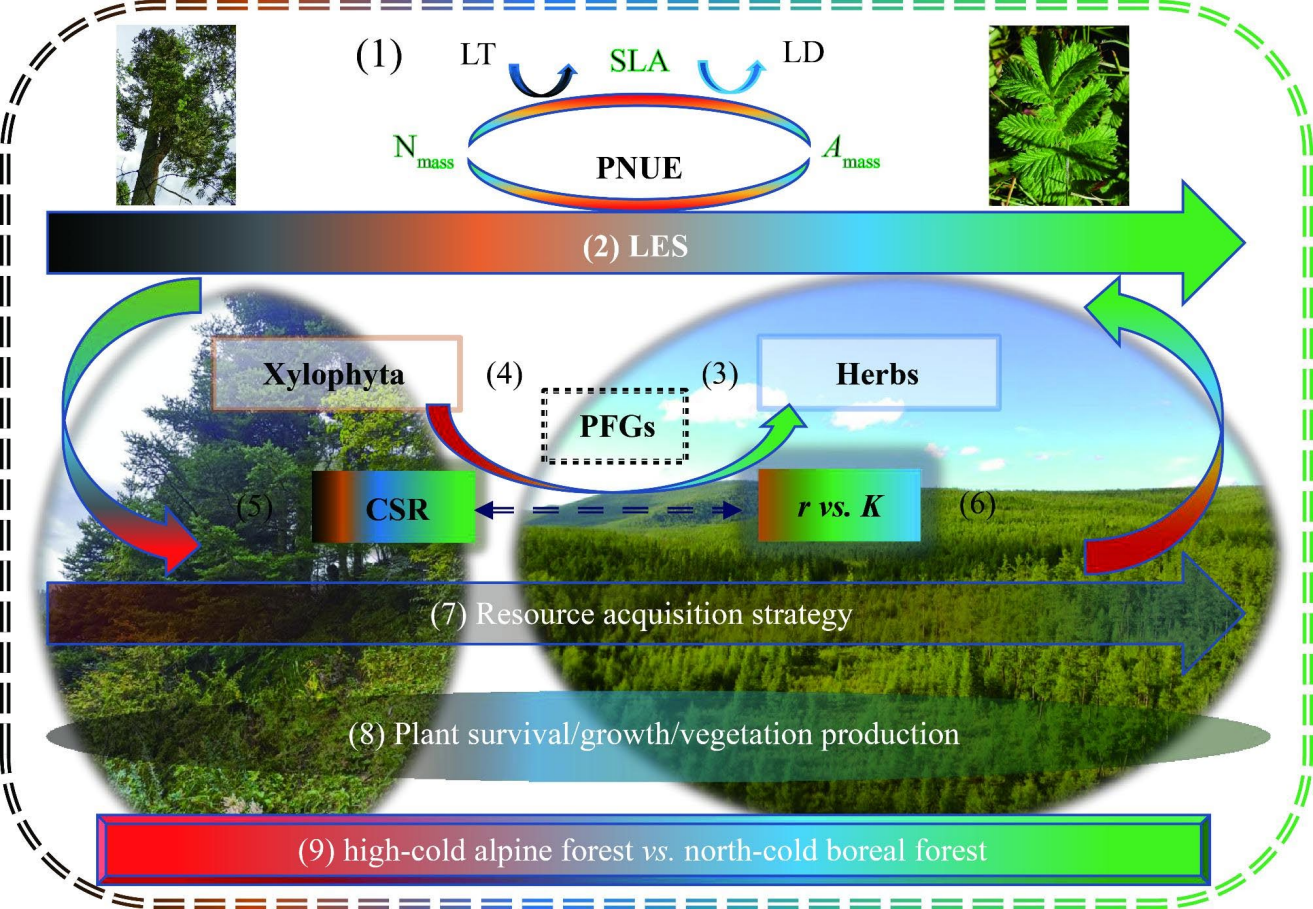
A conceptual model related to classical ecological theories based on coordination/balance among leaf functional traits in the two extremely harsh habitats. LES, leaf economics spectrum; PFG, plant functional group; CSR, Grime’s competitive–stress tolerant–ruderal triangle [105]; *r vs. K*, *r* - versus *K* - selection [107]. For other abbreviations (e.g., SLA), see Fig. 1

In summary, based on coordination/balance among leaf functional traits in the two extremely harsh habitats and the relevant previous studies, we roughly depicted the conceptual models related to classic ecological strategy theories (Fig. 8). The key leaf functional traits were selected to investigate their relationships, in which SLA is a core of the traits (1). The two endpoints of leaf economics spectrum (LES) (2) could be placed with herbs (3) and xylophyta (4), respectively. Herbs may tend to be placed at *R* angle side in Grime’s competitive–stress tolerant–ruderal (CSR) triangle [11, 105, 106] (5), and at *r*-endpoint in *r*- versus *K*-selection (*r vs. K*) [107] (6). Herbs seemed to be with resource acquisition strategy [68, 73] (7), particularly in boreal forest. Finally, plant survival/growth might be regulated via the synergistic/trade-off relationships between the traits (8). Together, they would be distinguished by the two extreme harsh habitats (high-cold alpine forest vs. north-cold boreal forest) (9).

## Conclusions

In the study, we analyzed the direct and indirect relationships between the key eight leaf functional traits related to leaf structure, nutrition status, and physiological activity, within a large number of plant species in both high-cold alpine forest vs. north-cold boreal forest. Most of the leaf functional traits at two sites were significantly correlated with each other, strongly corroborating their coordination and/or trade-offs in the two contrasting habitats. This largely proves that LES could appear in the two forests. The structural equation modeling revealed that leaf density had an indirect effect on PNUE, primarily mediated by leaf structure and photosynthesis. The resource acquisition strategy was observed in herbs rather than xylophyta across the two habitats, particularly in alpine forest. Finally, we found that the variations in leaf functional traits in north-cold boreal forest were largely distributed in the resource-acquisitive strategy spectrum, a quick investment-return behavior; while those in the high-cold alpine forest tended to be mainly placed at the resource-conservative strategy end. It implicates that the habitat specificity for the relationships between key functional traits might be determinant of local plant communities, which can roughly adhere to several classic ecological strategy theories (i.e., LES, CSR and *r vs.*. *K*). These findings can shed light on which and how plants adopt adaptive and evolutionary strategies to deal with long-term environmental factors, potentially assist to assess and project vegetation composition and functioning when facing climate change. Nevertheless, in the current study, the more environmental factors were not interacted with more functional traits in details such as photosynthetic processes. From a future perspective, it will be crucial to consider the photosynthetic pathway of plants and the combined effects of multiple environmental factors to forecast how the plant economic strategies deal with climate change in these contrasting plant communities at larger spatial-temporal scales.

### Methods

#### Site expressions

**Fig. 9:**
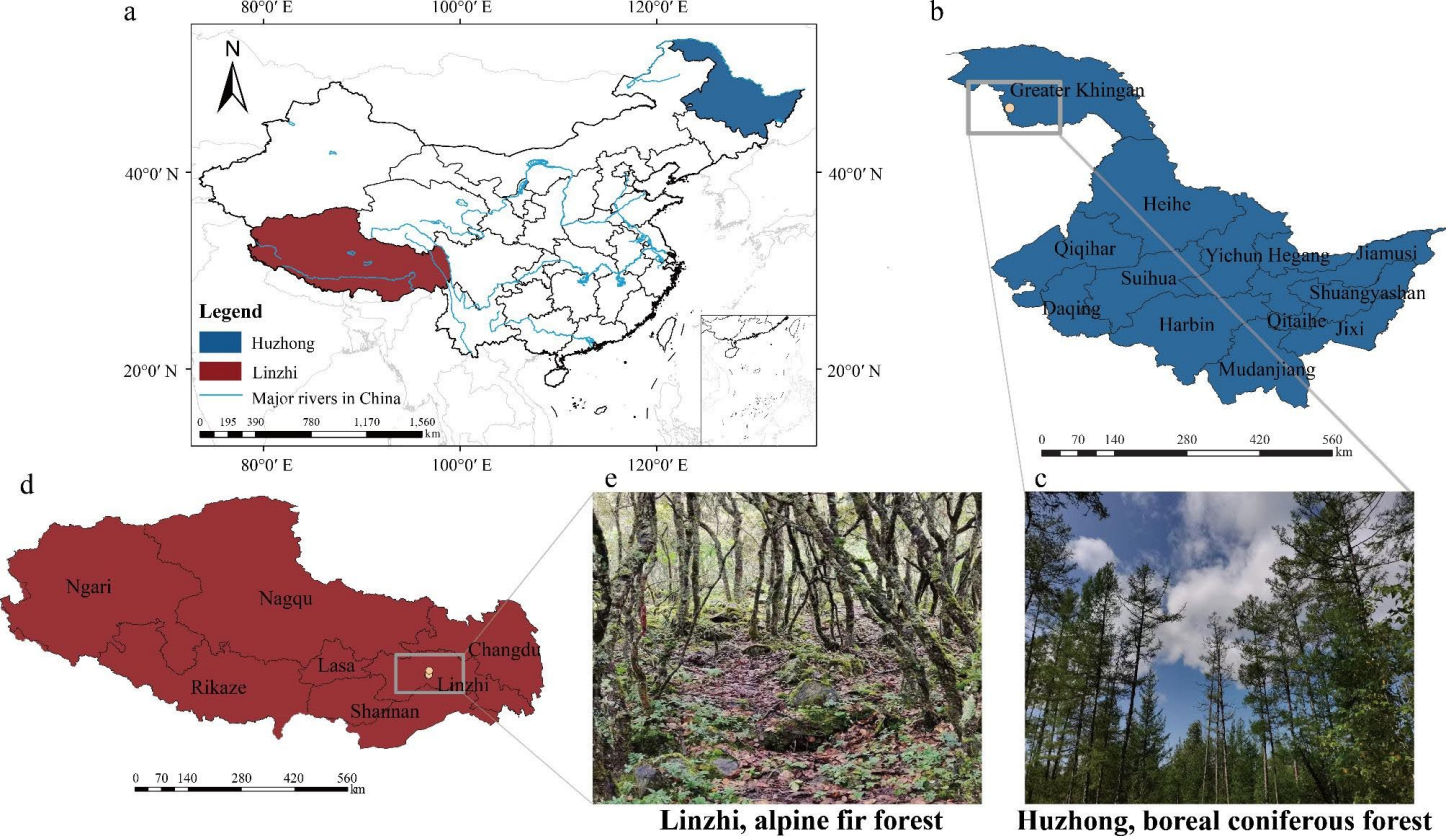
The study locations. (**a**) Map of China; (**b**) Huzhong site, Greater Khingan, Heilongjiang; (**c**) Boreal coniferous forest. (**d**) Linzhi site, Sygera Mountain, Tibet; (**e**) Alpine fir forest

The first study site is located in Sygera Mountain, Nyingchi (hereinafter referred to Linzhi) (29°39′ N-29°50′ N, 94°42′ E-94°44′ E), Tibet, China, with an altitude range from 3031 to 4300 m (Fig. 9). Records from the WorldClim2.1 dataset, from 1960 to 2018, show a mean annual precipitation (MAP) of 646 mm, of which 70.73% falls during the monsoon season (June to September, the major plant growth peaking period), and a mean annual temperature (MAT) of 3.91℃ (Table S8). The south Asian monsoon results in abundant summer rainfall, and the high altitude causes high-cold climate and intense ultraviolet radiation, which provides unique climatic characteristics [108]. The dominant vegetation in the area is alpine fir forest, and the dominant species include *Abies georgei* var. *Smithii* Cheng et L., *Potentilla xizangensis* Yü et Li, and *Quercus aquifolioides* Rehd. et Wils [108] (Table S9).

The second study site is located in Greater Khingan Range, Huzhong National Nature Reserve (hereinafter referred to Huzhong) (51°46′ N, 123°0′ E, 773 m a.s.l), Heilongjiang, Northeast China. The regional climate is represented by MAP of 537 mm and MAT of -4.57 °C over last 30 years (1960–2018). Around 80% of total annual precipitation occurs in plant growing season (June-September). July (mean temperature of 16.2℃) and January (-27.2℃) are the warmest and the coldest months of year, respectively (Table S1). The low temperature caused by high latitude is the characteristic in this area. The dominant vegetation is boreal coniferous forests (Fig. 9). The dominant species include *Larix gmelinii* Rupr., *Betula platyphylla* Suk., *Rhododendron simsii* Planch., and *Rhododendron tomentosum* Harmaja [99] (Table S9).

### Plot selection and sampling

From late July to later August in 2017 (Huzhong) and 2021 (Linzhi), when plants reached peak growth, we ensure the rigor of the research by collecting almost all species in undisturbed areas with minimal anthropogenic activities for visual inspection of the vegetation at both sites, and more than 3 mature individuals were randomly chosen. In this study, the selected species broadly are sorted into two contrasting climate types, i.e., high-cold alpine and north-cold temperate climates, respectively. Totally, 42 species belonging to 21 families and 37 genera were used to examine their leaf functional traits (Table S9). Of the total 42 species, 23 species were herbs and 19 were xylophyta (Table S9).

These leaf traits in 42 species in the two contrasting climate zones were measured (Table S1). As noted above, these traits selected might underlie differences in their growing environments and would have significant associations with the resource conservation/acquisition trade-off axis [16, 21, 33].

### Plant structural traits

A vernier caliper was used to measure the leaf thickness (LT) of each undamaged mature green leaf at its widest part to avoid the midrib [26]. Leaf samples were then scanned and calculated to leaf area using the ImageJ software [109]. Following these measurements, all leaf samples were then dried at 65 °C for at least 72 h to a constant weight, and weighed to determine dry matter weight. SLA was calculated as leaf area/leaf dry weight [110]. Leaf tissue density (LD) was calculated by the following equation:

1 $${\rm{LD }} = {\rm{ Leaf\, dry\, mass }}/\left( {{\rm{Leaf\, area \times LT}}} \right)$$

### Leaf nitrogen content and photosynthetic rate

Dried samples from each plant were ground using a ball mill. Leaf nitrogen concentration per unit mass (N_mass_) was determined with 8–9 mg of homogenously material for each sample using an elemental analyzer (Vario EL III, Elementar Analysensysteme Comp., Hanau, Germany). We obtained leaf nitrogen concentration per unit area (N_area_) using the following equation [111]:

2 $${{\rm{N}}_{{\rm{area}}}} = {{\rm{N}}_{{\rm{mass}}}}/{\rm{ SLA }} \times {\rm{ }}10$$

Leaf gas exchange parameters were measured using a CIRAS-2 portable photosynthesis system (PP Systems, Hertfordshire, UK) on clear sky mornings (9:00–11:00 a.m.) with less than gentle wind. The reference CO_2_ concentration in the leaf chamber was kept at 360–400 µmol CO_2_ mol^− 1^, with a relative air humidity of 50-70%, and saturating photosynthetic photon flux density was set at 1500 µmol·m^− 2^·s^− 1^. The fully expanded youngest leaves per plant per species in each plot were placed into the cuvette, and at least three measurements were made for each species in each plot [88]. The maximum net photosynthetic rate per unit area (*A*_area_) was directly obtained, and the maximum net photosynthetic rate per unit mass (*A*_mass_) was then calculated by the equation:

3 $${A_{{\rm{mass}}}} = {A_{{\rm{area}}}} \times {\rm{ SLA }}/10000$$

Finally, we calculated the photosynthetic nitrogen use efficiency (PNUE) using the formula [69]:

4 $${\rm{PNUE }} = {A_{{\rm{area}}}}/{\rm{N}_{{\rm{area}}}}$$

### Statistical analyses

All analyses were conducted with R version R-4.1.2 (R Development Core Team, 2021). First, the means and standard deviations (mean ± SD) for each trait were estimated to display contrasting values of the traits. Coefficient of variation (CV) by variance analysis was performed to test the differences in each trait between the plant functional types and climate types. We also estimated the associations between leaf functional traits by using the Spearman’s correlation analyses, which could reflect the proportion of variation in one variable that was accounted for by the variation in the other variable.

Regressions were performed to test the relationships between leaf nutrient (i.e., leaf N concentration), photosynthetic traits (i.e., net photosynthetic rate) and the leaf structural trait (i.e., SLA, LD) under each climate type. In detail, we tested the differences in the slopes and intercepts of regression lines at the two sites. A principal component analysis (PCA) was performed to visualize the distribution of two PFTs in the same climate type and test the difference in resource utilization strategies between alpine fir forest and boreal coniferous forest. Finally, a structural equation modeling (SEM) was used to test the direct and indirect effects between key leaf functional traits at the two sites. The indicators, such as the chi-squared test (Chisq > 0.05), goodness-of-fit index (GFI > 0.90) and root mean square error of approximation (RMSEA < 0.05), were used to test the models [112].

## Electronic supplementary material

Below is the link to the electronic supplementary material.

Supplementary Material 1

## Data Availability

The datasets used and analysed during the current study are available from the corresponding author on reasonable request.

## List of Abbreviations

LD: Leaf tissue density
LT: Leaf thickness
SLA: Specific leaf area
N_mass_: Leaf nitrogen concentration per unit mass
N_area_: Leaf nitrogen concentration per unit area
*A*_mass_: Light-saturated photosynthetic rate per unit mass
*A*_area_: Light-saturated photosynthetic rate per unit area
PNUE: Photosynthetic nitrogen use efficiency
