# Supplementary material for: Plant economic strategies in two contrasting forests
Source: BMC Plant Biol. 2023 Jul 22;23:366. doi: 10.1186/s12870-023-04375-9 (PMC10362557; doi:10.1186/s12870-023-04375-9)
Supplement: Supplementary file 1 — Supplementary Material 1 [file 12870_2023_4375_MOESM1_ESM.pdf]

Supplementary Materials

Title: Plant economic strategies in two contrasting forests
by Kuo Sun et al.

Table S1. Leaf functional traits measured in this study

Traits	Abbreviation	Units
Leaf tissue density	LD	$g \cdot cm^{-3}$
Leaf thickness	LT	mm
Specific leaf area	SLA	$cm^2 \cdot g^{-1}$
Leaf nitrogen concentration per unit mass	N_{mass}	$mg \cdot g^{-1}$
Leaf nitrogen concentration per unit area	N_{area}	$mg \cdot cm^{-2}$
Light-saturated net photosynthetic rate per unit mass	A_{mass}	$\mu mol \cdot g^{-1} \cdot s^{-1}$
Light-saturated net photosynthetic rate per unit area	A_{area}	$\mu mol \cdot m^{-2} \cdot s^{-1}$
Photosynthetic nitrogen use efficiency	PNUE	$\mu mol \cdot g^{-1}$ $N \cdot s^{-1}$

Table S2. Statistical summary on leaf functional traits among different plant functional types

Traits	Sites	Herb		Xylophyta		Total		Sig.
		Mean±SD	CV (%)	Mean±SD	CV (%)	Mean±SD	CV (%)	
LD ($g \cdot cm^{-3}$)	Huzhong	0.29±0.09Ba	29.54	0.38±0.13Aa	33.60	0.36±0.13	34.83	ns
	Linzhi	0.26±0.14Bb	53.53	0.84±0.52Aa	61.28	0.37±0.35	92.64	
LT (mm)	Huzhong	0.15±0.06Ab	36.79	0.16±0.09Ab	52.96	0.16±0.08	49.46	***
	Linzhi	0.36±0.14Aa	39.71	0.45±0.19Aa	42.94	0.38±0.16	41.42	
SLA ($cm^2 \cdot g^{-1}$)	Huzhong	272.51±102.05Aa	37.45	206.63±102.68Ba	49.69	224.60±105.93	47.16	*
	Linzhi	219.80±66.43Ab	30.23	62.20±32.76Bb	52.67	188.28±88.08	46.78	
N_{mass} ($mg \cdot g^{-1}$)	Huzhong	13.16±2.62Ab	19.93	13.96±5.76Aa	41.27	13.74±5.09	37.05	***
	Linzhi	28.57±7.92Aa	27.71	16.46±3.74Ba	22.72	26.02±8.77	33.68	
N_{area} ($mg \cdot cm^{-2}$)	Huzhong	0.59±0.33Bb	56.10	0.76±0.28Ab	36.94	0.72±0.30	42.41	***
	Linzhi	1.47±0.64Ba	43.32	3.26±1.59Aa	48.89	1.76±1.21	68.74	
A_{mass} ($\mu mol \cdot g^{-1} \cdot s^{-1}$)	Huzhong	0.11±0.06Bb	48.81	0.18±0.11Aa	59.92	0.16±0.10	62.21	ns
	Linzhi	0.18±0.08Aa	47.97	0.01±0.004Bb	30.83	0.16±0.09	60.77	
A_{area} ($\mu mol \cdot m^{-2} \cdot s^{-1}$)	Huzhong	4.18±1.15Bb	27.57	9.42±4.96Aa	52.68	7.99±4.86	60.90	ns
	Linzhi	8.37±4.06Aa	48.54	3.15±0.87Bb	27.77	7.76±4.18	53.92	
PNUE ($\mu mol \cdot g^{-1} N \cdot s^{-1}$)	Huzhong	8.80±3.94Ba	44.77	12.62±4.75Aa	37.61	11.58±4.83	41.68	***
	Linzhi	6.62±3.55Ab	53.61	0.75±0.17Bb	23.02	5.88±3.85	65.44	

Notes: Different capital and lowercase letters indicate significant differences among plant functional types and sites, respectively (p<0.05). Significant levels: ***, $p < 0.001$; **, $p < 0.01$; *, $p < 0.05$; ns, non-significance. For abbreviations, see Table S3.

Table S3. ANOVA on leaf functional traits at Huzhong and Linzhi sites

Traits		Df	Sum Sq	Mean Sq	F-value	*P*	diff	Sig.
LD	Hu×Lin	1	0.007	0.007	0.110	0.740	0.015	ns
($g·cm^{-3}$)	Residuals	124	8.120	0.065				
LT	Hu×Lin	1	1.536	1.536	101.800	0.000	0.221	***
(mm)	Residuals	124	1.871	0.015				
SLA	Hu×Lin	1	41458	41458	4.331	0.039	-36.320	*
($cm^2·g^{-1}$)	Residuals	124	1187050	9573				
N_{mass}	Hu×Lin	1	4616	4616	93.280	0.000	14.804	***
($mg·g^{-1}$)	Residuals	121	5988	49				
N_{area}	Hu×Lin	1	39.210	39.210	57.740	0.000	1.132	***
($mg·cm^{-2}$)	Residuals	121	82.180	0.680				
A_{mass}	Hu×Lin	1	0.002	0.002	0.184	0.646	-0.009	ns
($\mu mol·g^{-1}·s^{-1}$)	Residuals	115	1.131	0.010				
A_{area}	Hu×Lin	1	1.500	1.509	0.072	0.789	-0.229	ns
($\mu mol·m^{-2}·s^{-1}$)	Residuals	115	2413.300	20.985				
PNUE	Hu×Lin	1	901	901.000	45.650	0.000	-5.694	***
($\mu mol·g^{-1}N·s^{-1}$)	Residuals	112	2211	19.700				

Notes: Hu and Lin represent Huzhong and Linzhi sites, respective. Significant levels: ***, $p < 0.001$; **, $p < 0.01$; *, $p < 0.05$. For abbreviations, see Table S3.

Table S4. Statistical information on linear relationships between leaf function traits at Huzhong and Linzhi sites

Traits	Huzhong					Linzhi				
(X_Y)	Intercept	Slope	R^2	*F*	Sig.	Intercept	Slope	R^2	*F*	Sig.
SLA_LD	0.509	-0.001	0.318	29.827	0.000	0.917	-0.003	0.535	66.738	0.000
SLA_LT	0.263	-0.0005	0.386	40.285	0.000	0.386	-0.00003	0.000	0.014	0.906
SLA_N_{mass}	10.333	0.015	0.100	7.074	0.010	18.284	0.043	0.166	10.929	0.002
SLA_N_{area}	1.191	-0.002	0.545	76.732	0.000	3.791	-0.011	0.590	79.211	0.000
SLA_A_{mass}	0.071	0.0004	0.185	14.564	0.000	0.050	0.001	0.232	14.775	0.000
SLA_A_{area}	10.995	-0.013	0.085	5.947	0.018	7.706	0.0003	0.000	0.001	0.970
SLA_PNUE	7.319	0.019	0.173	13.409	0.001	0.443	0.028	0.357	25.588	0.000
LD_LT	0.194	-0.095	0.023	1.498	0.226	0.430	-0.132	0.085	5.379	0.024
LD_N_{mass}	16.251	-6.991	0.030	1.948	0.168	30.108	-10.556	0.179	12.026	0.001
LD_N_{area}	0.318	1.108	0.209	16.886	0.000	0.870	2.528	0.581	76.311	0.000
LD_A_{mass}	0.224	-0.165	0.041	2.714	0.104	0.207	-0.158	0.243	15.704	0.000
LD_A_{area}	4.380	10.040	0.067	4.576	0.036	8.665	-2.831	0.040	2.044	0.159
LD_PNUE	14.247	-7.426	0.037	2.465	0.121	8.011	-6.422	0.253	15.545	0.000
N_{mass}_N_{area}	-0.054	0.016	0.624	106.212	0.000	0.041	0.004	0.148	8.002	0.007
A_{mass}_A_{area}	1.045	9.699	0.366	36.998	0.000	9.216	-0.593	0.032	1.523	0.223

Notes: For abbreviations, see Table S3.

Table S5. Differences of slope and intercept of linear regression between two regions (Huzhong and Linzhi).

Traits(X_Y)	Difference in slope	Sig.	Difference in intercept	Sig.
SLA_LD	0.002	0.001	-0.408	0.001
SLA_N_{mass}	-0.028	0.001	-7.951	0.001
SLA_N_{area}	0.009	0.001	-2.600	0.001
SLA_A_{mass}	-0.001	0.001	0.157	0.021
SLA_PNUE	-0.009	0.001	6.876	0.001
LD_N_{area}	-1.420	0.001	-0.552	0.013
A_{mass}_A_{area}	0.012	0.001	-0.095	0.001

Notes: For abbreviations, see Table S3.

Table S6. Contribution rates of traits to PC1 and PC2 based on PCA analysis (%)

Traits	Huzhong		Linzhi		Total	
	PC1	PC2	PC1	PC2	PC1	PC2
LD	4.60	8.42	15.56	18.00	10.12	0.28
LT	11.81	2.87	0.002	11.10	7.40	10.23
SLA	18.03	14.14	15.36	14.11	17.38	1.31
N_{mass}	13.75	6.29	4.70	0.053	0.19	32.82
N_{area}	3.30	30.84	16.53	7.96	19.70	10.63
A_{mass}	25.30	6.50	20.13	11.54	17.43	18.77
A_{area}	4.10	29.66	9.64	32.39	4.72	25.96
PNUE	19.10	1.28	18.08	4.84	23.08	0.01

Notes: For abbreviations, see Table S3.

Table S7. Statistical information for structural equation modeling on the key leaf functional traits at both Huzhong and Linzhi sites

Sites	Chisq	df	*p*-value	GFI	CFI	RMR	SRMR	RMSEA	AIC	BIC
Huzhong	0.31	1	0.58	1.00	1.00	0.003	0.003	0.00	477.90	506.37
Linzhi	1.19	1	0.28	0.99	1.00	0.005	0.006	0.06	343.90	368.23

Notes: Chisq, Chisquare (χ^2); GFI, goodness-of-fit index; CFI, Bentler's comparative fit index; RMR, root mean square residual; SRMR, standardized root mean square residual; RMSEA, root mean square error of approximation; AIC, Akaike's information criterion; BIC, Bayesian information criterion.

Table S8. Climatic factors at both sampling sites (Huzhong, a north-cold boreal site; Linzhi, a high-cold alpine site)

Month	Average temperature/°C		Precipitation/mm		Solar radiation/kJ·m^{-2}·day^{-1}	
	Huzhong	Linzhi	Huzhong	Linzhi	Huzhong	Linzhi
Jan.	-27.2	-4.9	5.0	3.4	4212.0	11696.4
Feb.	-22.5	-3.5	5.0	7.2	7436.0	13277.6
Mar.	-14.5	-0.2	10.0	16.8	12439.0	15779.6
Apr.	-1.8	3.4	28.0	41.4	16465.0	18354.0
May.	6.5	7.2	39.0	68.0	19685.0	19641.2
Jun.	13.0	10.6	86.0	115.8	20983.0	17871.4
Jul.	16.2	11.6	138.0	131.6	19259.0	16718.6
Aug.	13.8	11.2	121.0	109.4	16226.0	16590.4
Sep.	6.7	9.4	69.0	100.4	12328.0	15642.4
Oct.	-3.4	5.4	19.0	41.0	8700.0	14411.0
Nov.	-16.3	0.2	11.0	8.2	4949.0	12947.4
Dec.	-25.1	-3.5	6.0	3.2	3303.0	11560.4
Total	-4.57	3.91	537.0	646.4	12165.4	15374.2

Notes: Huzhong, a north-cold boreal forest in Greater Khingan Range, Heilongjiang, Northeast China; Linzhi, a high-cold alpine forest in Sygera Mountain, Nyingchi, Tibet, China.

Table S9. Major plant species in this study (Huzhong, a north-cold boreal site; Linzhi, a high-cold alpine site)

Huzhong			Linzhi		
Species	Family	Growth from	Species	Family	Growth from
Aster tataricus	Compositae	Herb	*Abies georgei* var. *smithii*	Pinaceae	Xylophyta
Betula fruticosa	Betulaceae	Xylophyta	*Anemone rivularis*	Ranunculaceae	Herb
Betula platyphylla	Betulaceae	Xylophyta	*Carex* spp.	Cyperaceae	Herb
Calystegia silvatica	Convolvulaceae	Herb	*Cassiope selaginoides*	Ericaceae	Xylophyta
Carex spp.	Cyperaceae	Herb	*Cirsium eriophoroides*	Asteraceae	Herb
Duchesnea indica	Rosaceae	Herb	*Eleocharis yokoscensis*	Cyperaceae	Herb
Erysimum cheiranthoides	Cruciferae	Herb	*Fragaria nubicola*	Rosaceae	Herb
Filipendula palmata	Rosaceae	Herb	*Halenia elliptica*	Gentianaceae	Herb
Larix gmelinii	Pinaceae	Xylophyta	*Impatiens nyimana*	Balsaminaceae	Herb
Pinus pumila	Pinaceae	Xylophyta	*Parasenecio quinquelobus*	Asteraceae	Herb
Populus koreana	Salicaceae	Xylophyta	*Plantago asiatica*	Plantaginaceae	Herb
Pyrola asarifolia	Pyrolaceae	Xylophyta	*Polygonum capitatum*	Polygonaceae	Herb
Rhododendron dauricum	Ericaceae	Xylophyta	*Polygonum nepalense*	Polygonaceae	Herb
Rhododendron tomentosum	Ericaceae	Xylophyta	*Potentilla xizangensis*	Rosaceae	Herb
Ribes nigrum	Saxifragaceae	Xylophyta	*Primula alpicola*	Primulaceae	Herb
Rosa davurica	Rosaceae	Xylophyta	*Prunella vulgaris*	Labiatae	Herb
Salix matsudana	Salicaceae	Xylophyta	*Rubus* spp.	Rosaceae	Xylophyta
Sorbaria sorbifolia	Rosaceae	Xylophyta	*Sabina squamata*	Cupressaceae	Xylophyta
Spiraea salicifolia	Rosaceae	Xylophyta	*Senecio raphanifolius*	Asteraceae	Herb
Thalictrum aquilegifolium var. *sibiricum*	Ranunculaceae	Herb	*Tibetia himalaica*	Fabaceae	Herb
Vaccinium spp.	Ericaceae	Xylophyta			
Vaccinium vitis-idaea	Ericaceae	Xylophyta			
